# How Deep Brain Stimulation of the Nucleus Accumbens Affects the Cingulate Gyrus and Vice Versa

**DOI:** 10.3390/brainsci9010005

**Published:** 2019-01-04

**Authors:** Ioannis N. Mavridis

**Affiliations:** C.N.S. Alliance Research Group, 14123 Athens, Greece; pap-van@otenet.gr; Tel.: +30-697-832-7199

**Keywords:** anatomy, cingulate gyrus, deep brain stimulation, limbic circuits, neuropsychiatric disorders, nucleus accumbens

## Abstract

The nucleus accumbens (NA) and the cingulate gyrus (CG) are two vital limbic brain structures. They have attracted attention as deep brain stimulation (DBS) targets in the treatment of common refractory psychiatric illness. The primary purpose of this article was to review the current knowledge regarding the way that NA DBS affects the CG and vice versa. Methodologically, a thorough literature review was performed. According to the current literature, NA DBS modulates the function of several brain areas including the CG cortex. It specifically causes activation in the ipsilateral CG cortex and voltage-dependent reduction of its blood oxygenation. It also reverses anterior mid-CG cortex dysfunction and decreases metabolism in the subgenual CG. Moreover, NA DBS that induces mirth inhibits the function of the anterior CG cortex and enhances effective connectivity from anterior CG to the ventral striatum. On the other hand, although it is highly probable that CG DBS affects the NA, the exact nature of its effects remains unclear. Despite the increasing interest in psychiatric DBS, the available data on how NA DBS affects the CG and vice versa are restricted. This conclusion probably reflects the high complexity of the limbic circuits and necessitates further research.

## 1. Introduction

The nucleus accumbens (NA) is a major pleasure center of the brain and acts as a limbic-motor interface. It constitutes the major part of the ventral striatum and consists of two chemically distinct parts in humans (identified by immunostaining against specific dopamine and opioid receptors), a shell laterally and a core medially. The first is mainly connected to the limbic and the second to the extrapyramidal motor system [1,2,3,4]. The cingulate gyrus (CG), especially its anterior part, constitutes a bidirectional connection of the NA. More specifically, the NA core receives glutamatergic projections from the anterior CG cortex and the CG cortex is a direct and indirect (the subgenual CG cortex via the thalamus) efferent connection of the NA [1,5].

Additionally, both the NA and the CG cortex (its full extent) have connections with the subcallosal CG white matter [6], and both the anterior CG cortex and the NA send outputs to the medial tip of the subthalamic nucleus, which represents its limbic part [7]. The subgenual CG shows a wide range of connections with limbic, prefrontal, and mesiotemporal areas including the NA [8,9]. Moreover, the pregenual region of the anterior CG cortex is strongly connected to medial prefrontal and also to the anterior mid-CG cortex [10]. Interestingly, the CG cortex is probably the thickest cortical area connected to the NA [1,5].

Deep brain stimulation (DBS) has proven efficacy in neurobehavioral disorders, works by modulation of cortico–striato–pallido–thalamo–cortical circuits implicated in these disorders [10], and is emerging as a promising powerful tool for the alleviation of targeted symptoms in treatment-resistant neuropsychiatric disorders. The major targets of neuropsychiatric DBS, for treating disorders such as treatment-resistant depression (TRD) and obsessive-compulsive disorder (OCD), include the NA and the CG cortex (especially the subcallosal/subgenual CG/Brodmann area 25 [8,11,12,13]) or CG subgenual white matter [8,10,11,12,13,14,15,16,17,18]. These targets seem to be safe [13], with positive influence on mood and anxiety disorders and sometimes complete remission of the symptoms [15,16], although in a small number of cases [13].

Abnormal activity in cortico-striato-thalamo-cortical (fronto-striatal) circuits including the anterior CG cortex and ventral striatum has been implicated in the neuropathogenesis of compulsive disorders, such as OCD and anorexia nervosa [19,20]. DBS, e.g., of the NA, used for the treatment of OCD was initially thought to create a functional lesion as in ablative procedures. However, it is more probable that it may induce clinical benefit through activation of axonal fibers spanning the cortico-striato-thalamo-cortical circuits, alteration of oscillatory activity within this network, and/or release of critical neurotransmitters [20]. Furthermore, given the overlap in symptomatology and neurocircuitry between reward-related disorders such as such as major depression [21], OCD, and anorexia nervosa [19], and the established efficacy of NA DBS in OCD, it has been hypothesized that DBS of the NA and other areas associated with reward, e.g., the anterior CG cortex, might be an effective treatment for patients with chronic, treatment refractory anorexia nervosa [19]. Finally, Brodmann area 24a of the CG cortex [13] has been also reported as a potential DBS target for TRD [13], and the NA is also a promising target for the treatment of alcohol and heroin dependence [22].

As the subcallosal CG and NA are interconnected, one hypothesis is that by stimulating these targets one would just be influencing different relays in the same circuitry [15]. However, the issue arises as to whether NA DBS really affects the CG, and how. Conversely, could CG DBS have an impact on the NA? Searching for answers to these questions, the primary purpose of this article was to review the current knowledge regarding the way that NA DBS affects the CG and vice versa.

## 2. Materials and Methods

Methodologically a PubMed search for the terms “nucleus accumbens”, “deep brain stimulation” and “cingulate cortex” was performed and retrieved 42 results (1995–2018). The resulting relevant articles (only 24) formed the core of the materials used for this review. The criterion of relevance was the description of either accumbens DBS effect on CG or cingulate DBS effect on the NA. Consequently the resulting review is narrative in nature. No language limitations were noted. Additional data from experimental and clinical studies of the NA were also used and the functional role of the NA-CG connection was explored.

## 3. Results

### 3.1. Stimulation of the Nucleus Accumbens

Gibson et al. (2017) [23] studied OCD patients who underwent DBS of the ventral internal capsule/ventral striatum (affecting the NA) with intraoperative identification of localizations according to mirth-inducing and non-mirth-inducing stimulation. They found that only mirth-inducing DBS caused functional inhibition of the anterior CG cortex and that mirth-inducing DBS enhanced effective connectivity from this cortex to ventral striatum, while attenuating connectivity from thalamus to ventral striatum compared to non-mirth-inducing stimulation [23].

Knight et al. (2013) [17] utilized 3 Tesla functional magnetic resonance imaging (fMRI) and studied changes in a blood oxygenation level-dependent (BOLD) signal to test if NA/internal capsule DBS could result in global activation of brain networks in an animal model. They observed stimulation-evoked activation in the ipsilateral CG cortex among other cortical areas. Furthermore, as the stimulation voltage increased from 3 to 5 volts, the BOLD signal was decreased in the CG and prefrontal cortex. These results suggest that NA/internal capsule DBS causes modulation of psychiatrically important brain areas including the CG (as well as the prefrontal and insular) cortex [17].

Kuhn et al. (2011) [24] reported a patient with severe alcohol addiction who underwent NA DBS which resulted in normalization of craving and addictive behavior. They noticed that an electrophysiological marker of error processing (based on electroencephalographic recordings), linked to the anterior mid-CG cortex functioning, was altered through DBS, an effect that could be reversed in periods without stimulation. Thus, their case suggests that NA DBS may have a positive effect on addiction via normalization of craving associated with anterior mid-CG cortex dysfunction [24].

Bewernick et al. (2010) [25] reported 10 patients suffering from TRD (not responding to other treatments including electroconvulsive therapy) who were implanted with DBS electrodes bilaterally in the NA. Twelve months after the beginning of DBS treatment, five patients reached a 50% reduction of the Hamilton Depression Rating Scale. A significant increase was observed in the number of hedonic activities. Interestingly, ratings of anxiety (Hamilton Anxiety Scale) were reduced in the whole group but more boldly in the responders. Their [18F]-2-fluoro-2-deoxy-d-glucose positron emission tomography data revealed that NA DBS decreased metabolism in the subgenual CG among other (prefrontal) regions (including the orbital prefrontal cortex). Thus, NA DBS appeared to have antianxiety, antidepressant, and antianhedonic effects, which are correlated with localized metabolic brain changes [25].

Casquero-Veiga et al. [26] examined changes in brain glucose metabolism, weight gain and food intake after NA DBS in a rat model of obesity. They found that it caused increased metabolism in the CG cortex among other changes [26]. Pinhal et al. [27] applied DBS of the dorsal part of the ventral striatum in mice and found that it induced c-Fos expression around the electrode tip and in different regions of the prefrontal cortex, including the anterior CG cortex. However, this prefronto-cortical activation was more extensive when they targeted the internal capsule [27]. Additionally, Figee et al. [28] found that NA DBS normalized NA activity, reduced excessive connectivity between prefrontal cortex and NA, and decreased frontal low-frequency oscillations during symptom provocation in OCD patients [28].

Finally, it is worth mentioning that Dougherty et al. [29] reported a randomized controlled trial of DBS at the ventral capsule/ventral striatum (affecting the NA) for TRD, which did not demonstrate a significant difference in response rates between the active and control groups. In this case it seems that NA DBS did not work as expected and it remains unanswered if these results could be potentially affected by the connections between the NA and CG.

### 3.2. Stimulation of the Cingulate Gyrus

Chronic DBS of the subgenual CG white matter has led to remarkable remission of symptoms in some patients suffering from TRD [9]. The anatomical connectivity of the subgenual CG region, targeted stereotactically for electrode implantation in the treatment of depression, supports the theory that treatment efficacy is mediated by effects on a wide network of limbic, frontal, and visceromotor brain areas, including the NA [9]. However data regarding the nature of the effects of CG DBS on the NA are missing.

Real-time fMRI feedback in smokers has shown that modulation of the anterior CG cortex can decrease smokers’ craving for nicotine. Furthermore, decreased craving has been found in alcoholics after transcranial direct current stimulation or transcranial magnetic stimulation of the anterior CG cortex [22]. These results are similar to those from NA stimulation but the extent to which the NA contributes to the effects of the CG stimulation remains unclear.

Interesting experimental results by Vassoler et al. (2013) [30] showed that pharmacological inactivation of three medial prefrontal cortical areas including the anterior CG (the other two being the infralimbic and prelimbic cortices) weakened the reinstatement of cocaine seeking in a rat model. These results are similar with DBS of the NA shell attenuating cocaine reinstatement via local activation and/or activation of GABAergic interneurons in the medial prefrontal cortex through antidromic stimulation of afferent cortico-NA fibers [30]. Whether anterior CG inactivation through DBS could have similar effects remains an unanswered question.

### 3.3. Other Data

Metabolic depressions in the NA bilaterally and CG cortex, among other areas, have been elicited in rats by a unilateral electrolytic lesion of the centrolateral nucleus of the thalamus [31]. In addition, an extended and significant activation in the bilateral CG, as well as the NA, has been observed in patients with psychogenic erectile dysfunction who were treated with apomorphine [32]. Finally, prominent decreases of fMRI signals in regions including the NA and the anterior CG (Brodmann area 24) have been produced by acupuncture, with the opposite effect on the latter area when the subjects experienced pain [33].

## 4. Discussion

The CG is involved in regulation of emotional life, reactivity to painful stimuli, memory processing, and attention to sensory stimuli. Anatomically the CG cortex (as studied in monkeys) is composed of two distinct areas numbered 24 and 23 in Brodmann’s classification [34]. These two cingulate areas are interconnected and share several connections with deep gray matter and cortical areas. The anterior CG (Brodmann area 24) is particularly related to the intralaminar, mediodorsal, and ventral anterior thalamic nuclei, the amygdala, and of course the NA. [34]. The latter is implicated in a variety of forms of reward-related learning, reflecting its anatomical connections with limbic cortical structures including, as already mentioned, the CG [35].

The NA core, the anterior CG cortex, and the central nucleus of the amygdala are required for normal acquisition of tasks based on stimulus-reward associations [36]. Interesting and crucial functional anatomy information regarding these areas has been provided by experimental lesioning studies. Lesions of the anterior CG cortex, lesions of the NA core (and not NA shell) and also disconnection of the anterior CG cortex and NA core have been found to impair the acquisition of appetitive Pavlovian conditioning in an autoshaping procedure in rats. These findings show that the NA core and anterior CG cortex are “nodes” of a corticostriatal circuit involved in the learning process of reward stimuli [35]. On the other hand, lesions of the central nucleus of the amygdala do not impair animal performance in such stimulus-reward associated tasks. Interestingly a functional outcome difference between anterior CG cortex lesions and NA core lesions is that the first do not entirely abolish stimulus discrimination [36].

Both the anterior CG cortex and NA have been implicated in allowing animals to overcome effort constraints in order to obtain greater benefits [37]. Walton et al. [37] (2009), in their interesting rat experiments found that only lesions to the anterior CG cortex, and not 6-hydroxydopamine NA lesions, can cause a bias away from spending effort for greater reward when choosing between options of different competency [37]. Cardinal et al. [38] (2001) showed in rats that selective lesions of the NA core induce persistent impulsive choice (choosing a small or poor reward that is available immediately, in preference to a larger but delayed reward). Contrary to this finding, damage to the anterior CG cortex and medial prefrontal cortex (both being NA afferents), had no effect on this capacity. Remarkably, impulsive choice contributes to drug addiction, attention-deficit/hyperactivity disorder, mania, and personality disorders [38].

Regarding human research, Wacker et al. (2009) [39] used resting electroencephalography, fMRI, and volumetric analyses and found that anhedonia, but not other symptoms of depression or anxiety, was correlated with reduced NA responses to reward, reduced NA volume, and decreased resting activity (increased resting delta current density) in the rostral anterior CG cortex (an area implicated in positive subjective experience). Moreover, NA reward responses were inversely associated with rostral anterior CG cortex resting delta activity [39].

**Figure 1:**
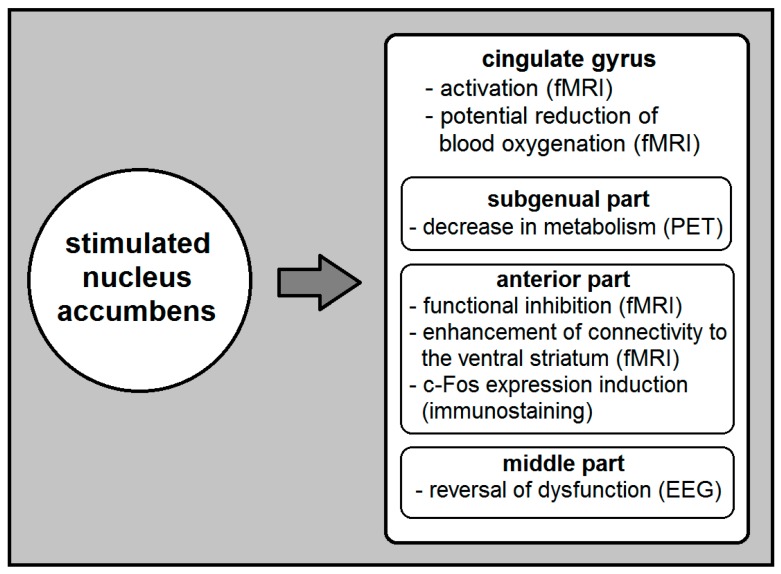
A simplified summary of the currently known effects of nucleus accumbens (NA) deep brain stimulation (DBS) on the cingulate gyrus (CG). The investigating method used to identify these effects appears in parentheses. EEG, electroencephalogram; fMRI, functional magnetic resonance imaging; PET, positron emission tomography.

According to the current literature, NA DBS modulates the function of several brain (primarily cortical) areas including the CG cortex (Figure 1). It specifically causes activation in the ipsilateral CG cortex, and voltage-depended reduction of its blood oxygenation [16]. It also reverses anterior mid-CG cortex dysfunction [23] and decreases metabolism in the subgenual CG [24]. Moreover, NA DBS that induces mirth inhibits the function of the anterior CG cortex and enhances effective connectivity from anterior CG to the ventral striatum [22]. On the other hand, although it is highly probable that the CG DBS affects the NA, the exact nature of its effects remains unclear.

Despite the increasing interest in psychiatric DBS and especially the role of the NA and CG as potential targets, the available data on how NA DBS affects the CG and vice versa are restricted. This observation depicts the high complexity of the limbic brain circuits and makes further research on this subject a modern necessity. Future studies are mandatory to determine the parameters of NA DBS that cause different CG parts to activate or deactivate, as well as how CG DBS could affect the NA and other vital limbic regions. The clinical significance of targeting different CG subregions for treating neuropsychiatric patients is also a field that needs further investigation.

## 5. Conclusions

The NA and CG are vital structures of the limbic brain circuits. They have attracted attention as potential DBS targets in the treatment of common refractory psychiatric illness. NA DBS may activate the CG cortex, reverse dysfunction of its middle part, and decrease metabolism in its subgenual part. It may also inhibit the function of its anterior part while enhancing its effective connectivity to the NA. In contrast, the exact effects of CG DBS on the NA remain unknown.
